# Associations between treatment burden, self-reported treatment qualities, antiretroviral therapy obtainment, and health-related quality of life among Ugandan PLWH

**DOI:** 10.1186/s12962-023-00434-y

**Published:** 2023-04-11

**Authors:** Ming Guan, Hongyi Guan

**Affiliations:** 1 Family Issues Center, Xuchang University, Xuchang City, Henan province China; 2 International Issues Center, Xuchang University, Xuchang City, Henan province China; 3 School of Business, Xuchang University, Xuchang City, Henan province China; 4 Middle School of Xuchang City, Grade 7 Class 18, No, Xuchang City, Henan province China

**Keywords:** Quality of life, Treatment burden, Self-reported treatment qualities, ART obtainment, PLWH, Uganda

## Abstract

**Background:**

Understanding related risk factors of health-related quality of life (HRQoL) could avoid treatment failure and provide an insight of personalized treatment approach among people living with HIV/AIDS (PLWH). The objective of this study was to identify factors associated with self-reported treatment qualities and domains of health-related quality of life (HRQoL) among PLWH in Uganda.

**Method:**

Data were from “Life on antiretroviral therapy: People’s adaptive coping and adjustment to living with HIV as a chronic condition in Wakiso District, Uganda” in English. The World Health Organization Quality of Life Brief Version (WHOQOL-BREF) questionnaire was used to assess the HRQoL of 263 PLWH in the sample. Considering variance inflation factors, multiple regression analyses were performed to assess the associations between demographic factors, ART obtainment, treatment burden, and self-reported treatment qualities, associations between demographic factors, self-reported treatment qualities, and HRQoL, and association between ART obtainment and HRQoL. Controlling for the confounding effects, several regression anatomies were employed to explore the associations between self-reported treatment qualities and six domains of HRQoL.

**Results:**

In the sample, the geographical distribution were urban (5.70%), semi-urban (37.26%), and rural (57.03%). 67.30% of the participants were females. The mean age of the sample was 39.82 years (standard deviation = 9.76) ranging from 22 to 81 years. Multiple logistic regressions reported statistically significant associations of distance to ART facility with self-reported quality of services, advice, manners, and counseling, statistically significant association between self-reported manners quality and four domains of HRQoL, and statistically significant association between TASO membership and domains of HRQoL. Plots from regression anatomies reported that self-reported treatment qualities had statistically significant associations with six domains of HRQoL.

**Conclusions:**

Treatment burden, self-reported treatment qualities, ART obtainment, and TASO were possible determinants of individual domains of HRQoL among PLWH in Uganda. PLWH’s HRQoL might be improved by promoting medical quality and optimizing ART obtainment in the healthcare providers’ practice. Findings in this study had important implications for the redesign of clinical guidelines, healthcare delivery, and health care co-ordination among PLWH globally.

## Background

In order to end HIV/AIDS by 2030, many innovative efforts have been developed to manage antiretroviral therapy (ART), health improvement, and health-related quality of life (HRQoL) among people living with HIV/AIDS (PLWH). Monitoring HRQoL and related risk factors among PLWH could identify treatment fatigue, treatment cancellation, treatment pause, treatment suspension, and therapy postponements in need of extra support and inform a personalized treatment approach. For instance, an international randomized trial discovered immediate treatment could significantly improve self-assessed quality of life (QoL) in healthy PLWH [1]. Another longitudinal study indicated QoL among PLWH had uniqueness in a clinical practice [2]. Nevertheless, current literature was scarce on studies that analyzed the relationship between treatment activities and adaptive adjustment among PLWH.

Recent studies underscored the psycho-social factors and disease-related factors in the HRQoL among PLWH during ART. For example, sociodemographic factors were strongly associated with lower HRQoL of PLWH [3, 4]. Meanwhile, social support from community [5, 6] and family [7] might improve HRQoL among PLWH. Recent research indicated that the presence of comorbidities continued to be prevalent symptoms and strongly associated with lower QoL among PLWH despite advances in HIV treatment in South East Nigeria [8]. Perceived stigma was reported to be correlated with the QoL [9], which led to poor adherence to active ART treatment regimen and subsequently resulted in treatment outcome among PLWH [10]. However, previous reports were limited to spirituality in the HRQoL and there was a lack of regarding associations between treatment-related factors and domains of HRQoL.

Prior research indicated that having HIV could lead to appreciable treatment and self-management burden. For patients, treatment burden was reflected by adherence to a prescribed, chronic condition self-management regimen [11], which included negative emotions and physical side effects [12], fragmented and poorly organized care [13], and economic burden [14, 15]. Theoretically, successful clinical decisions closely depended on a clinician’s ability to accurately gauge a patient’s treatment burden [16]. Empirically, a cross-sectional and secondary analysis demonstrated a subset of PLWH experienced high treatment burden related to chronic condition self-management [17]. Several studies reported ART traveling was associated with higher perceived treatment burden [18], clinical outcomes [19], and treatment fatigue [20]. Nevertheless, there was a lack of regarding associations between treatment burden and health outcomes among PLWH.

A substantial body of studies reported the methods to reduce treatment burden for PLWH [21–23]. As an example, TASO is an Ugandan non-governmental organization of HIV-infected and affected people in Uganda which is a country most affected by HIV/AIDS in the world. A cross-sectional study in Uganda concluded the burden of HIV infection in the medical emergency unit was high and majority of the patients who required ART had no prior HIV/AIDS care [24]. TASO provided counseling, AIDS information, nursing care, educational support, and material assistance to PLWH and their families at centres affiliated to district hospitals. TASO was confirmed to be ideal in caring for those affected by AIDS and HIV in Africa [25]. A retrospective cohort study also showed that good adherence and improved survival were feasible in TASO, Uganda [26]. Yet, further analyses are needed to determine whether TASO would be reformed.

As for PLWH, another important concept was treatment quality. The structural aspects of treatment quality could reflect operational quality in healthcare systems. For example, therapeutic relationship, provider characteristics, and treatment approach were identified as major dimensions of treatment quality by patients [27]. Another study in a psychiatric clinic indicated a higher staff consistency and staff density led to an improvement of treatment quality [28]. Likewise, a review found that current treatment for HIV still was accompanied by negative effects [29]. However, the associations of dimensions of treatment quality with domains of HRQoL among PLWH were seldom reported.

The literature as mentioned above showed ART treatment burden, self-reported treatment qualities, and ART obtainment had associations with HRQoL, respectively. Adherence to ART was reported to improve HRQoL among PLWH in not Ethiopia [30, 31] but Pakistan [32], Nigeria [33], and Brazil [34]. However, the the associations of interest were seldom reported in a Ugandan sample. The current study used a regionally representative sample from Uganda to explore the associations of interest on the basis of both descriptive and regression methods. Considering subjective treatment quality, confounding effects, and six domains of HRQoL, this study would possibly produce some new statistical outcomes compared to early studies.

## Methods

### Data source

This study used data from “Life on antiretroviral therapy: People’s adaptive coping and adjustment to living with HIV as a chronic condition in Wakiso District, Uganda” (Project code: RES-062-23-2663) with the questionnaire offered in English and Luganda [35]. The research in Uganda mainly analysed the experiences of PLWH following access to life-saving ART. The study aimed to understand PLWH’s adjustments to ART and to stimulate ART delivery policy and practice in medical, social and economic resource-constrained settings. The research involved collaboration between four partners: The School of International Development at University of East Anglia, and in Uganda the Medical Research Council, the AIDS Support Organisation, and a Ministry of Health hospital.

In this cross-sectional study, data were collected from November 1, 2010 to August 31, 2013. Three types of ART delivery site in Wakiso District were selected to recruit participants for the study: (1) the HIV clinic at the government hospital in Entebbe; (2) three government health centres (level 3) that have referral links to Entebbe, and (3) the Entebbe branch of a well-established non-governmental organisation, and The AIDS Support Organisation (TASO). A list of eligible patients (all those who had been on ART for at least one year) was compiled at each facility using patient files. With a systematic random sampling, the 263 PLWH were recruited randomly from the ART delivery sites using patient lists (patients on ART for less than 12 months were excluded). Considering the relationship to head of household, the respondents were head (66.54%), spouse (23.19%), parent (3.80%), sibling (2.66%), child (0.76%), other (2.66%), and not related (0.38%). The rural place (57.03%) was the main place where the respondents resided in, followed by semi-urban place (37.26%) and urban place (5.70%).

### Main variables

In this study, the scores of the HRQoL domains were scaled on the basis of Syntax file used to score the WHOQoL-BREF which was adopted on the website of Seattle Quality of Life Group [36]. Two early studies reflected the level of HRQoL assessed by the WHOQoL-BREF would improve the QoL for PLWH [37, 38]. Here, Cronbach alpha values and average interitem covariances of four domain items were computed. Cronbach alpha values of physical QoL domain items (f3, f4, f10, f15, f16, f17, and f18), psychological QoL domain items (f5, f6, f7, f11, f19, and f26), social QoL domain items (f20, f21, and f22), and environmental QoL domain items (f8, f9, f12, f13, f14, f2, f24, and f25) were 0.7523, 0.6748, 0.4200, and 0.7517, respectively. Average interitem covariances of physical QoL domain items (f3, f4, f10, f15, f16, f17, and f18), psychological QoL domain items (f5, f6, f7, f11, f19, and f26), social QoL domain items (f20, f21, and f22), and environmental QoL domain items (f8, f9, f12, f13, f14, f2, f24, and f25) were 0.3259, 0.2143, 0.2103, and 0.3076, respectively.

In this study, the six domains (physical QoL, psychological QoL, social QoL, environment QoL, general QoL, and general health) of the WHOQoL-BREF questionnaire were considered dependent variables. General QoL was measured by the question: “How do you rate your QoL?” Its response options were very poor (0.76%), poor (4.94%), fair (17.49%), good (57.79%), and very good (19.01%), respectively. Thus, poor general QoL was recorded as 1 (fair and below) and 0 (good or above). General health was measured by the question: “How satisfied with health?” Its response options were very dissatisfied (0.76%), dissatisfied (6.84%), fairly satisfied (9.51%), satisfied (61.22%), and very satisfied (21.67%), respectively. Thus, poor general health was defined as 1 (dissatisfied and below) and 0 (fairly satisfied or above). The other four domains (physical QoL, psychological QoL, social QoL, and environment QoL) of WHOQoL-BREF were conversed from Likert scale data into a 0–100 scale.

The independent variables considered in this study and their study-specific definitions were under ART obtainment and quality of service. Basically, they were demographic factors (age, sex, and enough meals daily), ART obtainment (number of daily pills, medicine change, ART Frequency, and ART duration), treatment burden (distance to ART facility, traveling time to ART facility, visiting cost to ART facility, and waiting time at ART facility), and self-reported treatment qualities (services quality, advice quality, manners quality, and counseling quality).

ART obtainment included number of daily pills, medicine change, ART frequency, and ART duration. Number of daily pills was measured by the question: “How many ART pills do you take per day?” The distribution of response options was 2 (84.03%), 3 (7.22%), 4 (7.98%), 5 (0.38%), and 8 (0.38%). Medicine change was measured by the question: “Have you had to change the medicine and move to a different type of ART?” Its response option of “same medicine as when started” was recoded as 0 and response option of “changed medicine” was recoded as 1. The response option of “don’t know” was treated as a missing value. ART frequency was measured by the question: “How frequently do you visit this ART provider?” The response options were once every week, once every two weeks, once every month, once every two months, and once every three months. Thus, the response options were recoded as 1 when ART frequency ≤ 1 time/month and as 0 when ART frequency > 1 time/month. ART duration was measured by the question: “For how long have you been obtaining ART from this facility?” The answer was calculated by the formula: 12×ART duration years + ART duration months.

Here, treatment burden was reflected by the workload of healthcare experienced by PLWH. Thus, according to the questionnaire of “Life on antiretroviral therapy”, the treatment burden was measured by several continuous variables including distance to ART facility, traveling time to ART facility, visiting cost to ART facility, and waiting time at ART facility. Distance to ART facility was measured by the question: “How far do you have to travel to the facility on the day you go to pick up the ART?” Its unit was mile. Travelling time to ART facility was reflected by the question: “How long does it take?” The response was calculated by a formula: 60×travelling hours + travelling minutes. Waiting time at ART facility was measured by the question: “How long do you usually have to wait at the facility on the day you go to pick up your medicine?” The response was calculated by a formula: 60×waiting hours + waiting minutes. Visiting cost to ART facility was measured by the question: “On your last visit how much did you spend on the journey to pick up your medicine?” The response was continuous value with unit of Uganda Shilling.

Self-reported services quality, self-reported advice quality, self-reported manners quality, and self-reported counseling quality were measured by the questions: “How would you describe / rate the overall quality of the services that you receive from the facility?”, “How would you rate the medical advice that you receive from the nurse / doctor?”, “How would you rate the manners or personal treatment that you receive from the nurse or doctor who you see at the facility?”, and “How would you describe the quality of the counseling services and advice that you receive from the nurse, doctor or HIV counselor?”, respectively. Their response options were 1 (= poor), 2 (= average/fair/OK), and 3 (= good) and were recoded as “below average” (0 = poor/average/fair/OK) and “above average” (1 = good).

### Statistical analysis

Since there were gender differences between HIV burden in terms of the life years lost [39], descriptive statistics were employed to describe the PLWH’s socio-demographic characteristics. The descriptive analyses included interquartile ranges and medians for continuous variables and frequencies and percentage for categorical variables. A chi-square test was used to determine the TASO membership difference in socio-demographic characteristics, HRQoL, treatment burden, self-reported treatment qualities, and ART obtainment separately. Thus, statistical significance of group difference could be judged by *p* value.

Several regression analyses were conducted to explore the targeted associations. First of all, multiple logistic regressions were employed to analyze association of demographic factors, ART obtainment, and treatment burden with self-reported treatment qualities. Secondly, multiple regressions were employed to analyze the associations of demographic factors and self-reported treatment qualities with HRQoL were conducted. Since physical QoL, psychological QoL, social QoL, and environment QoL were continuous variables, linear regressions were adopted. Simultaneously, logistic regressions were carried out because general QoL and general health were binary variables. And then, the similar approach was used to explore associations between ART obtainment and HRQoL. Finally, several regression anatomies [40] were constructed to explore association between self-reported treatment qualities and HRQoL when controlling for the confounding effects of sociodemographic factors and other treatment variables. Here, *p* value was also used to reflect statistical significance.

Multicollinearity in this study were diagnosed by stata program *collin* after the regression. As a rule of thumb, variance inflation factor (VIF) values of a group of variables were less than 10 might be unnecessary to merit further investigation. Thus, multicollinearity was not a problem and could be safely ignored.

The confidence interval and the significance level were denoted by 95% confidence interval (CI) and *p* value, respectively. Statistical analyses were done using Stata version 14.0 (STATA Corp., College Station, TX, USA).

## Results

### Characteristics of study population

A total of 263 respondents were studied, with mean age of 39.82 years (standard deviation = 9.76) ranging from 22 to 81 years. Among them, over half were females (67.30%). Considering food (in)security, they ate enough meals (87.07%) or went hungry (12.93%). The response options of ART frequency was once a fortnight (0.76%), once a month (55.89%), once every 2 months (41.44%), and once every 3 months (1.90%). Regarding facility types, they chose Grade A hospital (38.78%), Kasanje HC (III) hospital (10.65%), Nakawuka HC (III) hospital (3.80%), Kigungu (III) hospital (7.22%), TASO hospital (4.18%), and TASO outreach hospital (35.36%). Among the 263 patents, 63.50% respondents changed ART regimen, 36.12% did not changed, and 0.38% did not know whether the ART regimen was changed.

Table 1 reported demographic factors, treatment burden characteristics, and PLWH’s WHOQOL-BREF scores by TASO membership. There were significant statistical difference between TASO membership and non- TASO membership on enough meals daily, number of daily pills, medicine change, ART frequency, self-reported services quality, and psychological QoL.

**Table 1:** Demographic factors, ART process, treatment burden, self-reported treatment qualities, and HRQoL of the PLWH by TASO membership

Participant characteristics	TASO membership	Non-TASO membership	Total	Ch2	P value
**Demographic factors**					
Age (years), median (IQR)	40(34–48)	37(32–43)	38(32–45)	38.8929	0.727
Sex				0.0110	0.917
Female, N (%)	100(38.02)	77(29.28)			
Male, N (%)	48(18.25)	38(14.45)			
Enough daily meals				6.4732	0.011***
Yes, N (%)	122(46.39)	107(40.68)			
No, N (%)	26(9.89)	8(3.04)			
**ART obtainment**					
Number of daily pills, median (IQR)	2(2–2)	2(2–2)	2(2–2)	10.5323	0.032**
Medicine change				5.8017	0.016**
Yes, N (%)	103 (39.31)	64(24.43)			
No, N (%)	44(16.79)	51 (19.47)			
ART Frequency, N (%)				85.4363	0.000***
0, N (%)	101(38.41)	13(4.94)			
1, N (%)	47(17.87)	102 (38.78)			
ART duration (months), median (IQR)	48(33–72)	35(24–50)	41(25–60)	92.6183	0.108
**Treatment burden**					
Distance to ART facility, median (IQR)	2(1-5.5)	4(2–8)	3(1–7)	30.2091	0.113
Traveling time to ART facility, median (IQR)	40(22.5–90)	60(30–90)	60(30–90)	29.9075	0.419
Visiting cost to ART facility, median (IQR)	2000(1000–6000)	2000(1000–5000)	2000(1000–5000)	37.1675	0.325
Waiting time at ART facility, median (IQR)	180(120–240)	240(120–300)	180(120–300)	42.6188	0.122
**Self-reported treatment qualities**					
Self-reported services quality				3.3605	0.067*
Below average, N (%)	26(9.89)	31(11.79)			
Above average, N (%)	122(46.39)	84(31.93)			
Self-reported advice quality				1.0777	0.299
Below average, N (%)	11(4.18)	5(1.90)			
Above average, N (%)	137(52.09)	110(41.83)			
Self-reported manners quality				0.2072	0.649
Below average, N (%)	19(7.23)	17(6.46)			
Above average, N (%)	129(49.05)	98(37.26)			
Self-reported counseling quality				2.2297	0.135
Below average, N (%)	22(8.40)	10(3.82)			
Above average, N (%)	126(48.09)	104(39.69)			
**HRQoL**					
Physical QoL, median (IQR)	66.07 (53.57–71.43)	67.86(57.14-75.00)	67.86(57.143-75.00)	24.6806	0.214
Psychological QoL, median (IQR)	75(70.83–83.33)	79.17(75-87.50)	79.17(70.83–87.50)	23.0979	0.082*
Social QoL, median (IQR)	66.67(50.00–75.00)	66.67(58.33-75.00)	66.67(50.00–75.00)	8.4297	0.587
Environment QoL, median (IQR)	62.50(56.25–71.88)	65.63(56.25–71.88)	65.63(56.25–71.88)	22.7933	0.473
General QoL, N (%)	4(4–4)	4(4–4)	4(4–4)	3.5540	0.470
General health, N (%)	4(4–4)	4(4–4)	4(4–4)	4.2951	0.368

Note: *p < 0.10, **p < 0.05 and ***p < 0.01

### Association of demographic factors, ART obtainment, and treatment burden with self-reported treatment qualities

In Table 2, TASO membership had significant associations with self-reported counseling quality (aOR(adjusted odds ratio) = 0.28, 95% CI: 0.07–1.12). The respondents with enough meals daily were likely to report high advice quality (aOR = 3.31, 95% CI: 1.14–9.58). The respondents with ART frequency (≤ 1 time/month) were likely to report low advice quality (aOR = 0.35, 95% CI: 0.13–0.91) and low manners quality (aOR = 0.33, 95% CI: 0.09–1.22). ART duration had significant associations with self-reported advice quality (aOR = 1.03, 95% CI: 1.00-1.05). Distance to ART facility had significant associations with self-reported services quality (aOR = 1.09, 95% CI: 1.00-1.19), self-reported manners quality (aOR = 1.09, 95% CI: 1.00-1.18), and self-reported counseling quality (aOR = 1.13, 95% CI: 0.99–1.28).

**Table 2:** Associations of demographic factors, ART obtainment, and treatment burden with self-reported treatment qualities, AOR (95%CI)

	Self-reported services quality	Self-reported advice quality	Self-reported manners quality	Self-reported counseling quality	VIF
Age (years)	1.02(0.97–1.07)	1.01(0.95–1.07)	1.04(0.99–1.09)	1.04(0.98–1.10)	1.17
Sex (ref.: Female)					
Male	1.63(0.70–3.79)	1.27(0.36–4.48)	1.19(0.46–3.06)	2.36(0.77–7.20)	1.15
TASO member (ref.: No)					
Yes	0.85(0.32–2.28)	0.79(0.13–4.86)	0.68(0.20–2.34)	0.28*(0.07–1.12)	1.75
Enough daily meals (ref.: No)					
Yes	1.51(0.47–4.90)	3.31**(1.14–9.58)	1.54(0.52–4.62)	1.95(0.58–6.50)	1.05
Number of daily pills	0.94(0.55–1.63)	1.30(0.52–3.26)	0.96(0.51–1.84)	0.99(0.48–2.06)	1.10
Medicine change (ref.: No)					
Yes	1.77(0.83–3.76)	0.32(0.07–1.52)	0.99(0.44–2.20)	1.80(0.78–4.18)	1.24
ART Frequency (ref.:>1 time/month)					
≤ 1 time/month	0.35**(0.13–0.91)	0.60(0.10–3.72)	0.33*(0.09–1.22)	0.34(0.09–1.37)	1.82
ART duration (months)	1.00 (0.99–1.02)	1.03*(1.00-1.05)	1.01(0.99–1.03)	1.00(0.98–1.02)	1.18
Distance to ART facility	1.09*(1.00-1.19)	1.07(0.93–1.25)	1.09*(1.00-1.18)	1.13*(0.99–1.28)	1.63
Traveling time to ART facility	1.00(1.00-1.01)	1.01(1.00-1.02)	1.00(0.99-1.00)	1.00(0.99–1.01)	2.18
Visiting cost to ART facility	1.00(1.00–1.00)	1.00(1.00–1.00)	1.00(1.00–1.00)	1.00(1.00–1.00)	1.63
Waiting time at ART facility	1.00(1.00–1.00)	1.00(1.00–1.00)	1.00(1.00–1.00)	1.00(1.00–1.00)	1.09

Note: *p < 0.10 and **p < 0.05. AOR: adjusted odds ratio. VIF = Variance Inflation Factor. Mean VIF = 1.42

### Associations of demographic factors and self-reported treatment qualities with HRQoL

In Table 3, age, enough meals daily, and self-reported advice quality had significantly positive coefficients with physical QoL, psychological QoL, social QoL, and environment QoL, respectively. Male had significantly positive coefficients with psychological QoL. TASO membership had significantly positive coefficients with psychological QoL, social QoL, and environment QoL, respectively. Self-reported manners quality had significantly positive coefficients with environment QoL. Self-reported counseling quality had significantly positive coefficients with physical QoL and social QoL, respectively. Male respondents would be likely to have low general QoL (aOR = 0.20, 95% CI: 0.04–0.93). Enough meals daily had significant associations with general QoL (aOR = 0.22, 95% CI: 0.10–0.46) and general health (aOR = 0.26, 95% CI: 0.09–0.72).

**Table 3:** Associations of demographic factors and self-reported treatment qualities with domains of HRQoL, Coefficient (standardized errors) and AOR(95% CI)

Medical quality	Physical QoL	Psychological QoL	Social QoL	Environment QoL	General QoL	General health	VIF
Age	0.37*** (0.09)	0.65*** (0.09)	0.52*** (0.14)	0.49*** (0.09)	1.02(1.00-1.05)	0.98(0.95–1.02)	1.09
Sex (ref.: Female)							
Male	0.67(2.14)	4.98** (2.26)	-2.03(2.98)	1.60 (2.06)	0.70(0.36–1.38)	0.20**(0.04–0.93)	1.06
Enough daily meals (ref.: No)							
Yes	23.85*** (2.84)	17.40*** (3.00)	15.53*** (3.77)	21.31*** (2.73)	0.22***(0.10–0.46)	0.26**(0.09–0.72)	1.06
TASO member (ref.: No)							
Yes	3.12(2.01)	4.15* (2.12)	5.12* (2.87)	3.71* (1.93)	0.81 (0.44–1.49)		1.09
Self-reported services quality(ref.: ≤average)							
> average	-1.13(2.90)	-3.17(3.06)	4.28(4.14)	1.72(2.79)	0.86(0.39–1.91)		1.50
Self-reported advice quality(ref.: ≤average)							
> average	19.62*** (3.95)	34.10*** (4.17)	17.11*** (5.31)	16.45*** (3.80)			1.23
Self-reported manners quality (ref.: ≤average)							
> average	0.39(3.49)	-2.36(3.68)	0.57(4.64)	9.20*** (3.33)			1.51
Self-reported counseling quality (ref.: ≤average)							
> average	8.72** (3.47)	4.72(3.66)	10.10** (4.72)	-3.82(3.35)	0.61(0.24–1.52)	0.59 (0.19–1.83)	1.40
R-squared	0.9443	0.9572	0.9268	0.9468			
Adj R-squared	0.9425	0.9559	0.9235	0.9451			

Note: *p < 0.10, **p < 0.05 and ***p < 0.01. AOR = adjusted odds ratio. VIF = Variance Inflation Factor. Mean VIF = 1.24

### Association between ART obtainment and HRQoL

In Table 4, age, enough meals daily, number of daily pills, and medicine change had significantly positive coefficients with physical QoL, psychological QoL, social QoL, and environment QoL, respectively. Male gender had significantly positive association with psychological QoL (coefficient = 6.27, standardized error = 2.21) and significant association with general health (aOR = 0.18, 95% CI: 0.04–0.75), respectively. The respondents with enough meals daily would be likely to have low general QoL (aOR = 0.16, 95% CI: 0.07–0.34) and poor general health (aOR = 0.25, 95% CI: 0.09–0.65), respectively. TASO membership had significantly positive coefficients with physical QoL, psychological QoL, and environment QoL, respectively. ART frequency (≤ 1 time/month) had significantly positive coefficients with physical QoL, psychological QoL, and environment QoL and associations with general QoL (aOR = 2.01, 95% CI: 1.04–3.91), respectively. ART time (months) had significantly positive coefficients with physical QoL, social QoL, and environment QoL and significant associations with general QoL (aOR = 0.98, 95% CI: 0.97-1.00), respectively.

**Table 4:** Association between ART obtainment and HRQoL, Coefficient (standardized errors) and AOR(95% CI).

ART process	Physical QOL	Psychological QOL	Social QOL	Environment QOL	General QOL	General health	VIF
Age	0.35*** (0.09)	0.55*** (0.09)	0.59*** (0.15)	0.38*** (0.09)	1.00 (0.97–1.03)	0.97(0.93–1.01)	1.12
Sex (ref.: Female)							
Male	2.36(2.14)	6.27*** (2.21)	-2.15(3.02)	3.21 (1.99)	0.65(0.33–1.29)	0.18** (0.04–0.75)	1.07
Enough daily meals (ref.: No)							
Yes	23.34*** (2.91)	15.96*** (3.00)	15.54*** (3.88)	18.80*** (2.70)	0.16*** (0.07–0.34)	0.25*** (0.09–0.65)	1.07
TASO member (ref.: No)							
Yes	6.10** (2.36)	8.32*** (2.43)	4.55 (3.36)	4.81** (2.19)	1.22(0.64–2.31)	2.07(0.73–5.92)	1.57
Number of daily pills	4.59*** (1.25)	8.10*** (1.29)	5.09** (2.04)	6.35*** (1.16)	1.29(0.87–1.90)	0.93(0.46–1.90)	1.06
Medicine change (ref.: No)							
Yes	5.76*** (2.01)	7.88*** (2.07)	12.31*** (2.85)	7.10*** (1.86)	1.03(0.58–1.84)	1.30(0.50–3.35)	1.12
ART Frequency (ref.:>1 time/month)							
≤ 1 time/month	10.69*** (2.29)	13.60*** (2.36)	4.02 (3.30)	7.32*** (2.12)	2.01** (1.04–3.91)	1.19(0.47-3.00)	1.56
ART duration (months)	0.10** (0.04)	0.08* (0.05)	0.12* (0.07)	0.09** (0.04)	0.98* (0.97-1.00)	0.99(0.97–1.01)	1.14
R-squared	0.9445	0.9594	0.9247	0.9506			
Adj R-squared	0.9427	0.9581	0.9213	0.9490			

Note: *p < 0.10, **p < 0.05 and ***p < 0.01. AOR = adjusted odds ratio. VIF = Variance Inflation Factor. Mean VIF = 1.21

### Association between self-reported treatment qualities and HRQoL controlling for the confounding effects

**Fig. 1:**
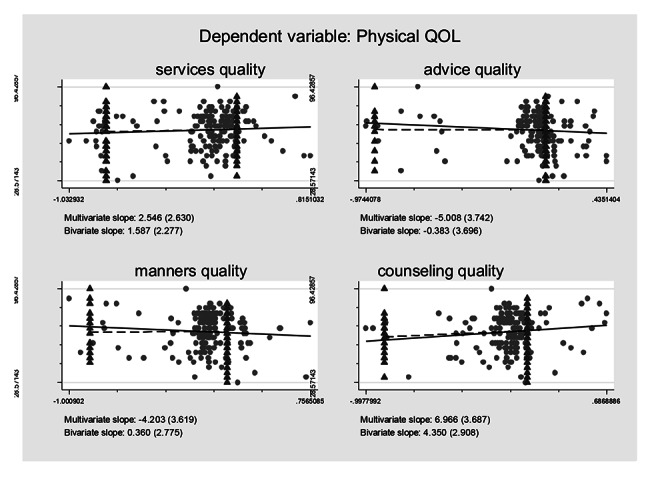
Composite graph of physical QOL Note: Regression lines: Solid = Multivariate, Dashed = Bivariate. Scatterplot: Dots = Transformed data, Triangles = Original data. VIFs of age, sex, enough meals daily, TASO membership, number of daily pills, medicine change, ART frequency, ART duration, distance to ART facility, traveling time to ART facility, visiting cost to ART facility, waiting time at ART facility, self-reported services quality, self-reported advice quality, self-reported manners quality, and self-reported counseling quality were 1.19, 1.17, 1.06, 1.78, 1.11, 1.32, 1.87, 1.19, 1.65, 2.22, 1.75, 1.13, 1.67, 1.31, 2.14, and 1.99. Mean VIF = 1.54

**Fig. 2:**
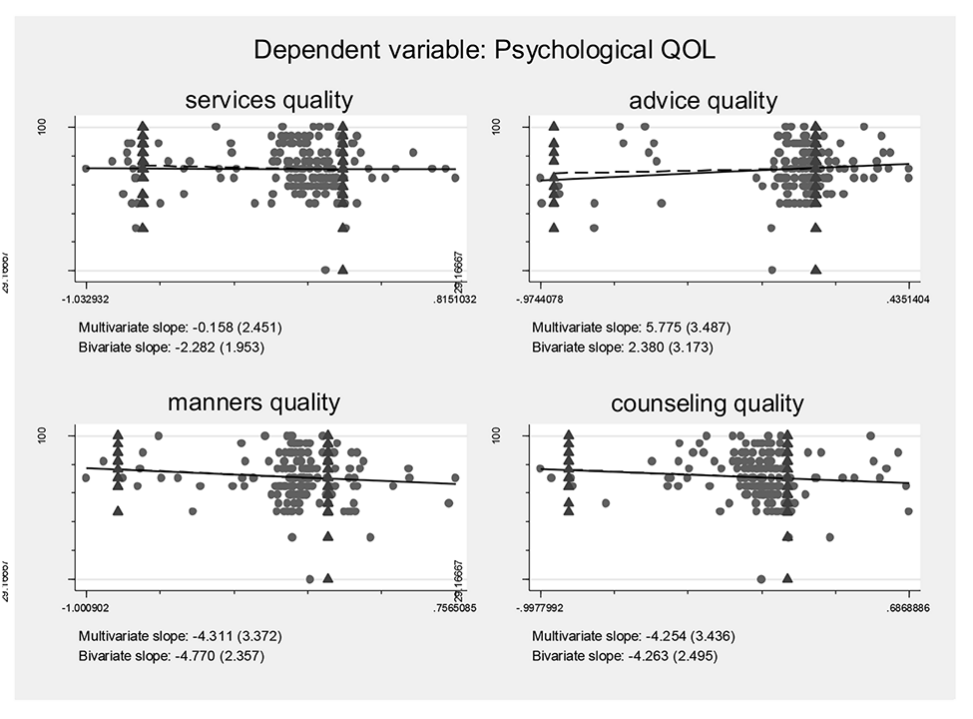
Composite graph of psychological QOL Note: Regression lines: Solid = Multivariate, Dashed = Bivariate. Scatterplot: Dots = Transformed data, Triangles = Original data. VIFs of age, sex, enough meals daily, TASO membership, number of daily pills, medicine change, ART frequency, ART duration, distance to ART facility, traveling time to ART facility, visiting cost to ART facility, waiting time at ART facility, self-reported services quality, self-reported advice quality, self-reported manners quality, and self-reported counseling quality were 1.19, 1.17, 1.06, 1.78, 1.11, 1.32, 1.87, 1.19, 1.65, 2.22, 1.75, 1.13, 1.67, 1.31, 2.14, and 1.99. Mean VIF = 1.54

**Fig. 3:**
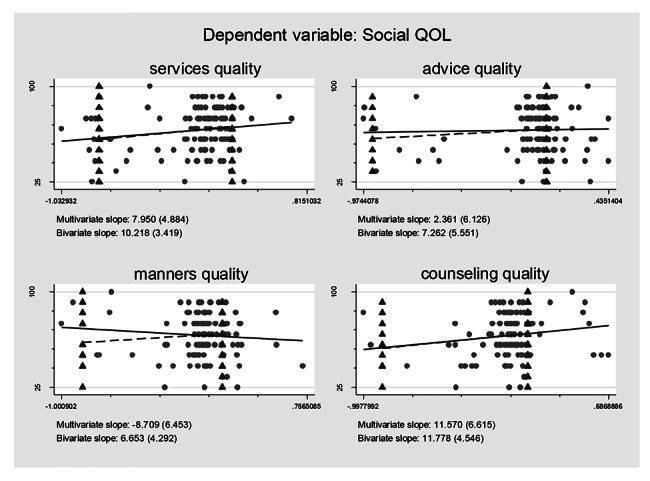
Composite graph of social QOL Note: Regression lines: Solid = Multivariate, Dashed = Bivariate. Scatterplot: Dots = Transformed data, Triangles = Original data. VIFs of age, sex, enough meals daily, TASO membership, number of daily pills, medicine change, ART frequency, ART duration, distance to ART facility, traveling time to ART facility, visiting cost to ART facility, waiting time at ART facility, self-reported services quality, self-reported advice quality, self-reported manners quality, and self-reported counseling quality were 1.19, 1.17, 1.06, 1.78, 1.11, 1.32, 1.87, 1.19, 1.65, 2.22, 1.75, 1.13, 1.67, 1.31, 2.14, and 1.99. Mean VIF = 1.54

**Fig. 4:**
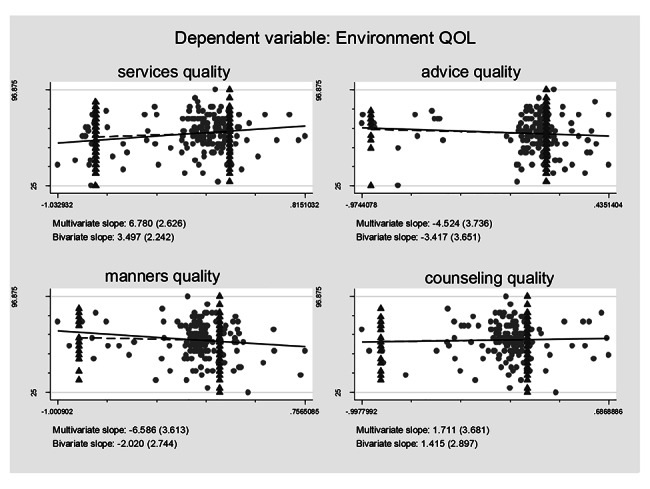
Composite graph of environment QOL Note: Regression lines: Solid = Multivariate, Dashed = Bivariate. Scatterplot: Dots = Transformed data, Triangles = Original data. VIFs of age, sex, enough meals daily, TASO membership, number of daily pills, medicine change, ART frequency, ART duration, distance to ART facility, traveling time to ART facility, visiting cost to ART facility, waiting time at ART facility, self-reported services quality, self-reported advice quality, self-reported manners quality, and self-reported counseling quality were 1.19, 1.17, 1.06, 1.78, 1.11, 1.32, 1.87, 1.19, 1.65, 2.22, 1.75, 1.13, 1.67, 1.31, 2.14, and 1.99. Mean VIF = 1.54

**Fig. 5:**
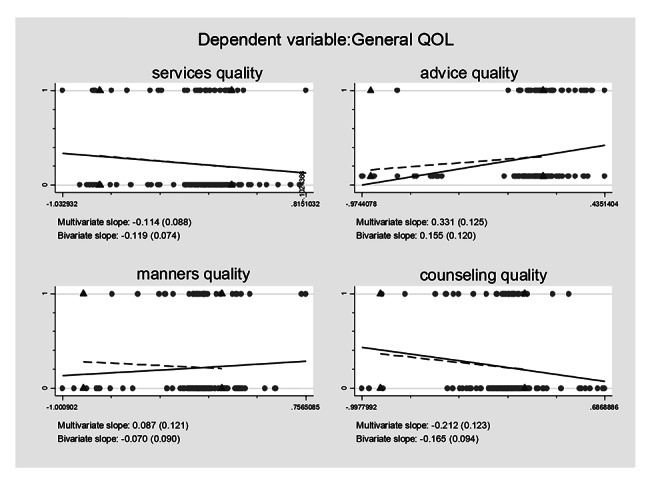
Composite graph of general QOL Note: Regression lines: Solid = Multivariate, Dashed = Bivariate. Scatterplot: Dots = Transformed data, Triangles = Original data. VIFs of age, sex, enough meals daily, TASO membership, number of daily pills, medicine change, ART frequency, ART duration, distance to ART facility, traveling time to ART facility, visiting cost to ART facility, waiting time at ART facility, self-reported services quality, self-reported advice quality, self-reported manners quality, and self-reported counseling quality were 1.19, 1.17, 1.06, 1.78, 1.11, 1.32, 1.87, 1.19, 1.65, 2.22, 1.75, 1.13, 1.67, 1.31, 2.14, and 1.99. Mean VIF = 1.54

**Fig. 6:**
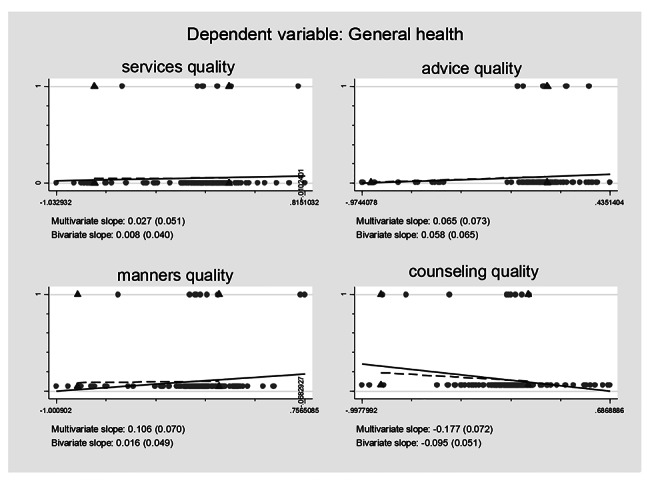
Composite graph of general health Note: Regression lines: Solid = Multivariate, Dashed = Bivariate. Scatterplot: Dots = Transformed data, Triangles = Original data. VIFs of age, sex, enough meals daily, TASO membership, number of daily pills, medicine change, ART frequency, ART duration, distance to ART facility, traveling time to ART facility, visiting cost to ART facility, waiting time at ART facility, self-reported services quality, self-reported advice quality, self-reported manners quality, and self-reported counseling quality were 1.19, 1.17, 1.06, 1.78, 1.11, 1.32, 1.87, 1.19, 1.65, 2.22, 1.75, 1.13, 1.67, 1.31, 2.14, and 1.99. Mean VIF = 1.54

Controlling for the confounding effects of treatment burden/ART obtainment on the self-reported treatment qualities and their effects on the HRQoL, multiple logistic regression models including all the self-reported treatment qualities variables were constructed based on regression anatomies and plotted by Figs. 1, 2, 3, 4, 5 and 6, respectively. Figure 1 showed self-reported services quality, self-reported manners quality, and self-reported counseling quality had positive associations with physical QoL, respectively, while self-reported advice quality had negative associations with physical QoL on the basis of bivariate slope. Figure 2 showed self-reported services quality, self-reported manners quality, and self-reported counseling quality had negative associations with psychological QoL, respectively, while self-reported advice quality had positive associations with psychological QoL in the case of bivariate slope. Figure 3 showed self-reported services quality, self-reported advice quality, and self-reported counseling quality had positive associations with social QoL, respectively, while self-reported manners quality had negative associations with social QoL under the condition of bivariate slope. Figure 4 showed self-reported services quality and self-reported counseling quality had positive associations with environment QoL, respectively, while self-reported advice quality and self-reported manners quality had negative associations with environment QoL in the term of bivariate slope. Figure 5 showed self-reported advice quality had positive associations with general QoL, while self-reported services quality, self-reported counseling quality, and self-reported manners quality had negative associations with general QoL with regard to bivariate slope, respectively. Figure 6 showed self-reported services quality, self-reported manners quality, and self-reported counseling quality had negative associations with general health, respectively, while self-reported advice quality had positive associations with general health with respect to bivariate slope. These results were not in line with an investigation which emphasized that HIV treatments did not negatively impact QoL [41].

## Discussion

### Summary of principal findings

The findings from this study informed the associations between treatment burden, self-reported treatment qualities, ART obtainment, and HRQoL in Ugandan PLWH. To our best knowledge, it was the first real-world study in Uganda that demonstrated ART frequency and distance to ART facility were associated with self-reported treatment qualities, respectively. The present findings also showed that age, enough meals daily, self-reported advice quality (> average value), medicine change, ART frequency (≤ 1 time/month), and ART time (months) were associated with domains of HRQoL, respectively. This study confirmed treatment burden played an important role in self-reported treatment qualities.

### Key explanations

With unique preferences and HRQoL, PLWH need optimal patient-centered care from health care providers. This was supported by a prior study that quality-of-care satisfaction significantly predicted treatment adherence [42]. Seemingly, increase in distance to ART facility could improve the self-reported services quality, manners quality, and counseling quality. Intuitively, this could reflect that ART system could provide high-quality care for PLWH. Perhaps simultaneously, PLWH prioritized his/her arrangement of clinical visits, and ART obtainment, treatment acceptance when assessing self-reported treatment qualities. In order to improve HRQoL among PLWH, those associations of interest should be analyzed on the basis of relevant literature.

The associations between demographic factors and ART obtainment was in congruence with a number of early studies. For example, age at diagnosis was directly associated with the type of treatment chosen [43]. Prior studies also had noted the importance of distance to ART facility. For instance, several prior studies found distance to health facility influenced uptake of prevention regimens [44], high mortality [45], and visiting times [46]. A study conducted in rural Malawi suggested that reduction in travel distance could achieve the ART coverage and increase access to ART [47]. Although there was a need for interventions to promote physical activity in the later ART treatment phases [48], increased physical activity was not associated with improvement in overall HIV symptom burden [49] or HRQoL [50] among PLWH. Regarding starvation, a systematic review and meta-analysis concluded macronutrient supplementation at ART initiation might positively influence immunologic response among PLWH in Sub-Saharan Africa [51]. Thus, it was important to improve socioeconomic status among PLWH.

This study confirmed medicine change played an important role in ART, which was in agreement with previous studies. Medically, Cihlar and Fordyce (2016) reported the key points of regimen selection for combination ART [52]. Evidently, antiretroviral adherence and treatment fatigue had been inextricably linked [53]. Likewise, several new drugs [54] had been developed with low toxicity [55] to replace the poorly effective drugs. In addition, a study conducted in Nigeria observed effectiveness, safety and tolerability appeared unaffected by the ART changes [56]. A hospital based retrospective study also concluded moving away from drugs with poor safety profiles could reduce modification rates and improve regimen tolerability [57]. In practice, once-daily antiretroviral schedules appeared to be as effective as twice-daily dosing regimens, with better adherence and treatment satisfaction [58].

Remarkably, treatment burden was found to have association with self-reported treatment qualities in the present study. This could be explained by an early study that distance to HIV care might be associated with retention in care and viral suppression [59]. Similarly, a study in Nigeria indicated that the high cost of transportation, HIV stigma, and long waiting hours were found to be key barriers to the use of ART services [60]. Another remarkable finding was that traveling time to ART facility, visiting cost to ART facility, and waiting time at ART facility were not associated with domains of HRQoL, which could be explained by several prior studies. For example, a prospective cohort study argued for realistic interventions and policy changes designed to reduce the financial and time burden of ART and to reduce logistical barriers [61]. Likewise, treatment burden could be influenced by micro and macro organization of health services [62] and the quality and configuration of health and social care services [63]. More often than not, self-efficacy was associated with health behavior and medication adherence among PLWH. Also, another study suggested a need for intervention programs to improve self-efficacy for disease management and QoL among PLWH [64].

This study also highlighted the role of treatment burden in ART. From a macro perspective, the finding in the present study was in line with several scholars who criticized that the over-burdened health system would not be able to maintain services quality [65]. Clinically, ART medication adherence was important factors associated with PLWH’s QoL [66]. Thus, long-term treatment burden could be interpreted as a mirror of self-reported treatment qualities. Furthermore, the existence of treatment burden could explain why technology-mediated interventions [67], psychosocial interventions [68], and community-based navigator intervention [69] could not improve overall QoL but improve clinical effectiveness in outcomes among PLWH. Regarding treatment effectiveness, the same factors with treatment burden were financial stresses and fear of HIV stigma [70], health care providers [71], and their responsibilities [72] in improving QoL for PLWH.

Regarding insignificant association between number of daily pills and domains of HRQoL, a possible explanation for this might result from side effects [73] due to the social burden of the epidemic [74]. Furthermore, an empirical review indicated various pill burdens per day would produce adherence differences [75]. This could be a possible explanation for appropriate adherence to ART. Adherence to treatment might in part be impacted by the circumstances that the individual brought to the treatment behavior [76]. Especially, there was a low adherence in the previous 30 days among PLWH in a rural area of China [77]. However, poor adherence to ART was associated with less effective viral suppression [78]. In order to solve the problem, several prior studies proposed designs of community-based adherence support [79], alleviated patient costs [80], and minimized pill burden with convenient formulations [81]. In particular, most of the QoL attributes were important to ART choice and could be used to optimize adherence and satisfaction among PLWH [82]. However, nursing interventions [83, 84] and focus on the real needs [85] could improve QoL of PLWH.

Here, TASO had significant associations with part of self-reported treatment qualities and domains of HRQoL. The most plausible reason for the associations has been discussed in previous studies. With respect to policy effectiveness, TASO was confirmed to better PLWH’s QoL [86, 87]. But, many TASO workers currently were infected with HIV or had AIDS [88]. TASO had shown that specialized services to meet AIDS care needs could be added to existing health services at district levels [89]. Another similar study in Wakiso District, Uganda reported PLWH on ART had significantly higher QoL for physical, psychological and environment domains, but not the social domain [90]. A retrospective longitudinal study also suggested that increased treatment burden was associated with greater health-care-resource utilization and high overall costs [91]. Still, a study revealed that psychosocial activities indirectly reduced treatment burden in chronically ill patients [92]. Thus, TASO need be changed and reformed.

Regarding associations between self-reported treatment qualities and six domains of HRQoL, this study encouraged us to analyse international successful experiences and rethink ART system in Uganda. At the nation-level, interventions to improve dietary diversity and food security in Ghana [93], the US AIDS drug assistance program [94], and lifestyle modification program in Tehran/Iran [95] had the potential to improve HRQoL among PLWH. But, a systematized review demonstrated mental health interventions for PLWH in low and middle-income countries were lack of efficacy [96]. Also, the U.S. National HIV/AIDS Strategy was questioned with respect to cost-effective manner [97]. These treatment approaches could not meet the personalized needs of PLWH or potentially minimized costs to already overburdened health care systems. Regarding many individuals newly infected with HIV, national-level strategy could improve the QoL among PLWH [98]. Still, two studies in France and Indian highlighted social support in improving QoL among PLWH [99, 100]. However, in some African countries, for example, the disclosure of HIV status to the community was very low in Southwest Ethiopia [101]. Therefore, some effective micro interventions could compete against mainstream national-level health care policies in the HIV/AIDS-affected areas.

### Strengths and limitations

Two strengths of this study were noteworthy. First, this study provided important information on the treatment among PLWH in Uganda, a population that was increasing but has received limited research attention. Second, a broad range of potentially associated treatment factors were explored, which had rarely been considered in the prior literature for ART.

This study was limited in the following areas. First, limited by the design of the original questionnaire, nearly all of the variables in this study were patient-centred. Thus, the treatment burden was estimated on the basis of PLWH rather than from both physicians’ and nurses’ perspective. Moreover till now, there was still a need to develop a standard and validated assessment method to measure treatment burden [102, 103]. Second, the sample of this cross-sectional study was dominated by female in gender. Therefore, it was important to be cautious with respect to the generalizability of the study findings. Possibly, the both PLWH genders experienced distinctly the treatment burden regarding visits to doctors, medical tests, treatment management, and lifestyle changes.

## Conclusion

In conclusion, treatment burden, self-reported treatment qualities, ART obtainment, and TASO membership were possible contributors to change in HRQoL among PLWH in Uganda. Self-reported quality of services, advice, manners, and counseling could be likely to be changed by distance to ART facility. New reform strategies could be enlightened by the statistical outcomes. Thus, it suggested Uganda should reform TASO membership, attenuate ART treatment burden, and optimize ART systems further.

## Data Availability

http://reshare.ukdataservice.ac.uk/851094/.

## Abbreviations

aOR: adjusted odds ratio
95% CI: 95% confidence interval
PLWH: people living with HIV/AIDS
QoL: quality of life
ART: antiretroviral therapy
HRQoL: health-related quality of life
WHOQOL-BREF: World Health Organization Quality of Life Brief Version
